# Effects of αTAT1 and HDAC5 on axonal regeneration in adult neurons

**DOI:** 10.1371/journal.pone.0177496

**Published:** 2017-05-15

**Authors:** Shen Lin, Noelle A. Sterling, Ian P. Junker, Courtney T. Helm, George M. Smith

**Affiliations:** Center for Neural Repair and Rehabilitation, Department of Neuroscience, Shriners Hospitals for Pediatric Research Center, Temple University School of Medicine, Philadelphia, Pennsylvania, United States of America; Imperial College London, UNITED KINGDOM

## Abstract

The role of posttranslational modifications in axonal injury and regeneration has been widely studied but there has been little consensus over the mechanism by which each modification affects adult axonal growth. Acetylation is known to play an important role in a variety of neuronal functions and its homeostasis is controlled by two enzyme families: the Histone Deacetylases (HDACs) and Histone Acetyl Transferases (HATs). Recent studies show that HDAC5 deacetylates microtubules in the axonal cytoplasm as part of an injury-induced regeneration response, but little is known about how acetylation of microtubules plays a role. Alpha-tubulin acetyl transferase (αTAT1) is a microtubule specific acetyl transferase that binds to microtubules and directly affects microtubule stability in cells. We hypothesize that increasing tubulin acetylation may play an important role in increasing the rate of axonal growth. In this study, we infected cultured adult DRG neurons with αTAT1 and αTAT1-D157N, a catalytically inactive mutant, and HDAC5, using lentiviruses. We found that αTAT1 significantly increases tubulin acetylation in 293T cells and DRG neurons but αTAT1-D157N does not. Furthermore, in neurons infected with αTAT1, a significant increase in acetylated tubulin was detected towards the distal portion of the axon but this increase was not detected in neurons infected with αTAT1-D157N. However, we found a significant increase in axon lengths of DRG neurons after αTAT1 and αTAT1-D157N infection, but no effect on axon lengths after infection with HDAC5. Our results suggest that while αTAT1 may play a role in axon growth *in vitro*, the increase is not directly due to acetylation of axonal microtubules. Our results also show that HDAC5 overexpression in the axonal cytoplasm does not play a crucial role in axonal regeneration of cultured DRG neurons. We expressed these genes in DRG neurons in adult rats and performed a sciatic nerve crush. We found that axons did not regenerate any better when infected with any of the constructs compared with control animals. Thus, while αTAT1 may be important for axonal growth *in vitro*, neither αTAT1 nor HDAC5 had an effect *in vivo* on the regeneration of sciatic nerves.

## Introduction

Histone acetyl transferases (HATs) and histone deacetylases (HDACs) carry out acetylation and de-acetylation respectively and are involved in many homeostatic cellular functions including cell division, cell growth and proliferation [1, 2]. These acetylation enzymes are also important in brain function, such as axon and dendrite development, memory formation, synaptic plasticity and the prevention of neurodegenerative diseases [3, 4]. Few studies have looked at how acetylation affects axonal growth in adult neurons after axonal injury. Promotion of histone acetylation in the nucleus by broadly upregulating HATs or downregulating the HDAC family proteins has previously been shown to increase axonal growth and neuronal migration [5–9]. However, experiments testing non-specific inhibitors of HATs and HDACs for their effects on axonal regeneration have sometimes yielded mixed results, especially when different concentrations are used [10]. The regulation of microtubule stability by acetylation and by other forms of posttranslational modification could play a role in axonal regeneration [11, 12]. Stabilized long-lived microtubules are correlated with higher levels of acetylation at Lysine 40 while unstable shorter-lived microtubules are less acetylated, and exhibit higher levels of tyrosination [13, 14].

Impairment of microtubule deacetylation by knockdown or inhibition of cytoplasmic HDAC5 and HDAC6 can inhibit axonal growth [15–17]. In neurons, axotomy-induced calcium influx is thought to be a key signal in the export of HDAC5 from the nucleus to the axonal cytoplasm [15, 16]. HDAC5 in the axonal cytoplasm is correlated with a graded increase in microtubule deacetylation towards the distal axon and this is thought to be important for sensory axonal regeneration [15, 18]. On the other hand, little is known about the effects of alpha-tubulin acetylases on axonal growth. One acetylation enzyme has been shown to directly acetylate microtubules *in vitro* and *in vivo*, namely alpha-tubulin acetyl transferase (αTAT1 / MEC-17), [19–24]. Knockdown of αTAT1 results in decreased microtubule acetylation and reduced sperm motility [19]. In C. elegans MEC-17 is responsible for mechanosensation of touch sensitive neurons [22, 24]. Recent studies also show that knockdown of αTAT1 results in loss of sensitivity to touch and pain stimulation in mice [25]. However, little is known about the mechanism of αTAT1 function in neuronal grown and regeneration. Although αTAT1 can acetylate itself through auto-acetylation, it is thought to have higher affinity for Lysine 40 tubulin acetylation, on the luminal side of microtubules [20]. Mutation of the αTAT1 auto-acetylation site, at aspartate 157 (αTAT1-D157N), results in loss of microtubule acetylation but does not affect binding affinity for alpha-tubulin [20, 21].

To further elucidate the role that acetylation plays in axonal regeneration we tested the effects of overexpressing HDAC5, αTAT1 and the αTAT1 catalytically inactive mutant (αTAT1-D157N) in adult DRGs *in vitro* and *in vivo*. We found that although αTAT1 increases alpha-tubulin acetylation in cultured DRG neurons and acetylation the axon, αTAT1-D157N does not have such an effect. However, we found that both αTAT1 and αTAT1-D157N significantly increase axonal lengths of DRG neurons *in vitro*. We also found that HDAC5 has a minimal effect on axonal regeneration either *in vitro* or *in vivo*, regardless of whether it is located in the cell nucleus or in the axonal cytoplasm. In sciatic nerves the overexpression of these constructs do not have a significant effect on axonal regeneration.

## Materials and methods

### Animals

Sprague Dawley rats (50 g, 21 days old, Harlan) were killed and DRG neurons were dissected for *in vitro* studies following previous protocols [10, 26]. A total of 22 adult female Sprague Dawley rats (200-225g; Harlan) were used in this study in surgical procedures. All surgical interventions and postoperative animal care were provided in accordance with the guide for the care and use of laboratory animals, and the guidelines for rodent survival surgery provided and approved by the Institutional Animal Care and Use Committees of Temple University.

### Lentiviral vector construction and production

pCSC-SP-PW-GFP, aka GFP-pBOB (GFP) lentivirus vector was acquired from Addgene (#12337). To generate αTAT1-pBOB and αTAT1-D157N-pBOB viral plasmids, the template vectors were acquired from Addgene (αTAT1: #27099 and αTAT1-D157N: #27100). These plasmids as well as the HDAC5 constructs all encode a GFP tag for easy identification of transduced cells. The following primers were used to amplify the PCR product of αTAT1 and αTAT1-D157N from the DNA template: GFP Forward Primer, 5'-CCTCTAGAGCCACCATGGTGAGCAAGGGC-3', αTAT1 Reverse Primer: 5'-TGGGATCCTCATTAGTATCGACTCTCCTCAGAGCG-3'. The PCR products were ligated to the XbaI/BamHI sites on the pBOB vector. The pBOB vector contains the CAG promoter to drive the expression of αTAT1 and αTAT1-D157N. Two HDAC5 mutant lentiviral constructs HDAC5-Cyt and HDAC5-Nuc [16], were kindly provided by Dr. Valerie Cavalli (University of Washington, St Louis, MO USA). All DNA constructs were verified by sequencing (GENEWIZ, South Plainfield, NJ). Viral stocks were generated by calcium phosphate transfection of 293T cells with plasmid encoding GFP, HDAC5, αTAT1 or αTAT1-D157N. Each plasmid was also transfected with Mb1, Rev and VSV-G plasmid to encode the viral envelope and glycoprotein. The supernatants were collected 72 hrs after transfection and concentrated by ultracentrifugation before the viral pellet was aliquoted and stored at −80°C until further use. The titers of GFP, HDAC5, αTAT1 and αTAT1-D157N lentivirus were measured by using p24 ELISA DIY Kit (Aalto, Ireland).

### DRG neuronal cultures

Sprague Dawley rats (50 g, 21 days old, Harlan) were killed and DRGs were dissected before mechanical dissociation and plating on poly-d-lysine and laminin coated 35 mm dishes [10]. Neurons were left to grow overnight before infection with lentiviruses for GFP, HDAC5, αTAT1 or αTAT1-D157N the next day. Cultures were grown for another 2 days in order for viral infection to take effect. Following this, the infected neurons were replated onto 22 x 40 mm coverslips and left to grow for 24 hrs and 31 hrs. For cultures infected with HDAC5, neurons were incubated in Ingenol-3-Acetate (I3A, Sigma) for 2 hrs at the time of replate before the drug was washed off and fresh media was added. For immunocytochemistry, the neurons were fixed with 4% paraformaldehyde with glutaraldehyde in PHEM buffer (60 mM PIPES, 25 mM HEPES, 10 mM EGTA, 2 mM MgCl_2_) for 10 min before blocking with 10% goat serum and 1% BSA in PBS for 30 min and permeabilized with 0.1% Triton X-100. Fixed cells were incubated with monoclonal anti-beta-III-tubulin antibody (1:1000, Promega), anti-GFP antibody (1:1000, Aves Labs) acetylated alpha-tubulin, 6-11B-1 (1:200, Sigma) or alpha-tubulin (1:200, Thermofisher) at 4°C overnight. Next day, the samples were rinsed three times with PBS before incubation with Texas red-anti-rabbit IgG (1:400, Jackson ImmunoResearch, West Grove, PA) and FITC-anti-mouse IgG (1:400, Jackson ImmunoResearch, West Grove, PA) at room temperature for 60 min. Images were taken of neurons labeled by beta-III-tubulin and GFP or acetylated tubulin, alpha-tubulin and GFP. For quantification of neurite outgrowth, the length of all neurites for the first 30 neurons encountered was determined by tracing, using the Neuron J plugin (www.ImageScience.org) of ImageJ analysis software. To quantify the ratio of acetylated alpha-tubulin to total alpha-tubulin along the axon length, axons between 100–200 μm were imaged and the fluorescence intensities of acetylated alpha-tubulin were ratioed against total alpha-tubulin. Each axon measured was divided into 10 equal segments and the ratios were plotted for each construct. Fluorescence intensities were determined using Carl Zeiss Axiovision LE Rel 4.4 software.

### Western blotting

DRG neurons and 293T cells were harvested from culture dishes after infection with lentiviruses for GFP, αTAT1 or αTAT1-D157N. The cells were lysed in 0.5% Triton X-100, 3% SDS, 50 mM Tris-HCl, pH 7.4, 150 mM NaCl, and 1 mM EDTA containing protease inhibitors and phosphatase inhibitor. After blocking in 5% milk for 1 hr at room temperature, the blots were incubated with anti-acetylated tubulin 6-11B-1 (1:1000, Sigma), anti-beta-III-tubulin (1:500, Promega) and anti-alpha-tubulin (1:10,000, Thermofisher) overnight at 4°C. The corresponding conjugated second antibodies donkey anti-rabbit IRDye-800CW and goat anti-mouse IRdye-680CW (1:20,000; LI-COR Biosciences) were added the next day and incubated for 1 hr at room temperature before being washed three times for 15 mins in TBS-Tween. Fluorescent blots were imaged on the Odyssey Infrared Imaging System (LI-COR Biosciences). The densitometric ratio of each band was quantified using the LI-COR Image Studio 3.1 software. To look at the acetylation levels of cells, the densitometric ratio of acetylated alpha-tubulin was ratioed to total alpha-tubulin for each sample. The ratio of samples infected with GFP was set as “1” and ratio value of samples infected with αTAT1 or αTAT1-D157N were compared to it.

### Surgeries

A total of 22 adult female Sprague Dawley rats (200-225g; Harlan) were used in this study. The rats were anesthetized by intraperitoneal injection of ketamine (67 mg/kg) and xylazine (6.7 mg/kg). A small incision was made in the skin along the posterior thigh to expose the gluteus muscle. The muscle was separated to expose the sciatic nerve. The sciatic nerve was injected using a 30s Hamilton Syringe with 1% lysolecithin (Sigma), following an adapted protocol described in Zhang et al., 2010 [27]. The nerve was injected at 3 points (1 ul each) separated by ~0.5 cm intervals and the syringe was left in the nerve for 2 mins after each injection point to allow the lysolecithin to diffuse in. The muscle was closed in layers and the skin was closed with staples. After each surgery, the rats were placed on a temperature controlled heating pad to allow the animal to recover. Rats were allowed to recover for 5 days before injection of lentiviruses for GFP, HDAC5, αTAT1 or αTAT1-D157N (1 μl of virus was injected at each of 3 points separated by 0.5 cm intervals). Following injection of the virus the rats were left for 3 weeks before the sciatic nerve was crushed. Using #5 Dumont forceps, a triple crush lesion of 10 secs was inflicted at a site on the sciatic nerve close to the injection points. The forceps were dipped in graphite powder before use in order to accurately mark the site of injury at the sciatic nerve. For animals injected with HDAC5, 5 mM PMA (Phorbol Myristate Acetate) was injected subcutaneously above the L4/L5 DRG to stimulate HDAC5 shuttling into the axonal cytoplasm [15, 18]. PMA is a PKC activator which phosphorylates HDAC5 and transports it to the cytoplasm [28].

### Tissue processing and immunohistochemistry

Six days after sciatic nerve crush, animals were sacrificed by injection of Fatal-Plus (Dearborn, MI) and perfused transcardially with 0.9% NaCl, followed by 4% paraformaldehyde (PFA) in 0.1 M phosphate buffer (PB, pH 7.4). The lesioned sciatic nerve was harvested and all tissues were post-fixed in 4% PFA at 4°C overnight before being moved to 30% sucrose in 0.1 M PBS at 4°C for 2–3 days. Tissue blocks were embedded in M-1 Embedding Matrix (Kalamazoo, MI) and quick frozen on dry-ice. The sciatic nerves were longitudinally sectioned at 20 μm using a cryostat and mounted directly on slides (Superfrost Plus; VWR International). DRGs were cut from the dorsal roots and also sectioned at 10 μm. For staining, the sections were permeabilized and non-specific antigenic sites blocked with 0.3% Triton X-100/10% normal goat serum in 0.1 M PBS for 1 hr at room temperature and then were incubated with chicken anti-GFP (1:1000, Aves Lab) and mouse anti-SCG10 antibody (1:1000, Novus), at 4°C overnight. Next day, the samples were rinsed three times, incubated with Texas red-anti-rabbit or FITC-anti-chicken IgG, at room temperature for 90 mins. After staining, sections were coverslip-mounted with Fluoromount-G (SouthernBiotech, San Diego, CA) and photographed using a Zeiss microscope. To examine axonal regeneration after sciatic nerve crush, we counted the number of axons positive for GFP, SCG10 or both GFP and SCG10 at various distances before and after the crush site. Samples were taken at 5 mm before the crush site and 1 mm, 3 mm, 5 mm, 7 mm, 10 mm and 12 mm distal to the crush site. A regeneration index was calculated based on the percentage of total axons measured across all the distances for each site for GFP, SCG10 or both GFP and SCG10.

### Statistical analysis

Raw data were processed, statistical analyses were performed, and graphs and charts were produced using Graphpad Prism 6. All data for image analysis on axon lengths and Western blotting densitometric quantifications were tabulated and statistical analysis was done using one-way analysis of variance (ANOVA) followed by the Tukey post hoc test to determine significant differences between groups. For statistical analysis of acetylated tubulin ratio along axons and of axon counts in the sciatic nerve, a two-way analysis of variance was done. Data represent the mean ± SEM. P values below the 5% probability level were considered significant.

## Results

### αTAT1 acetylates tubulin in 293T cells and in adult DRG neurons

**Fig 1:**
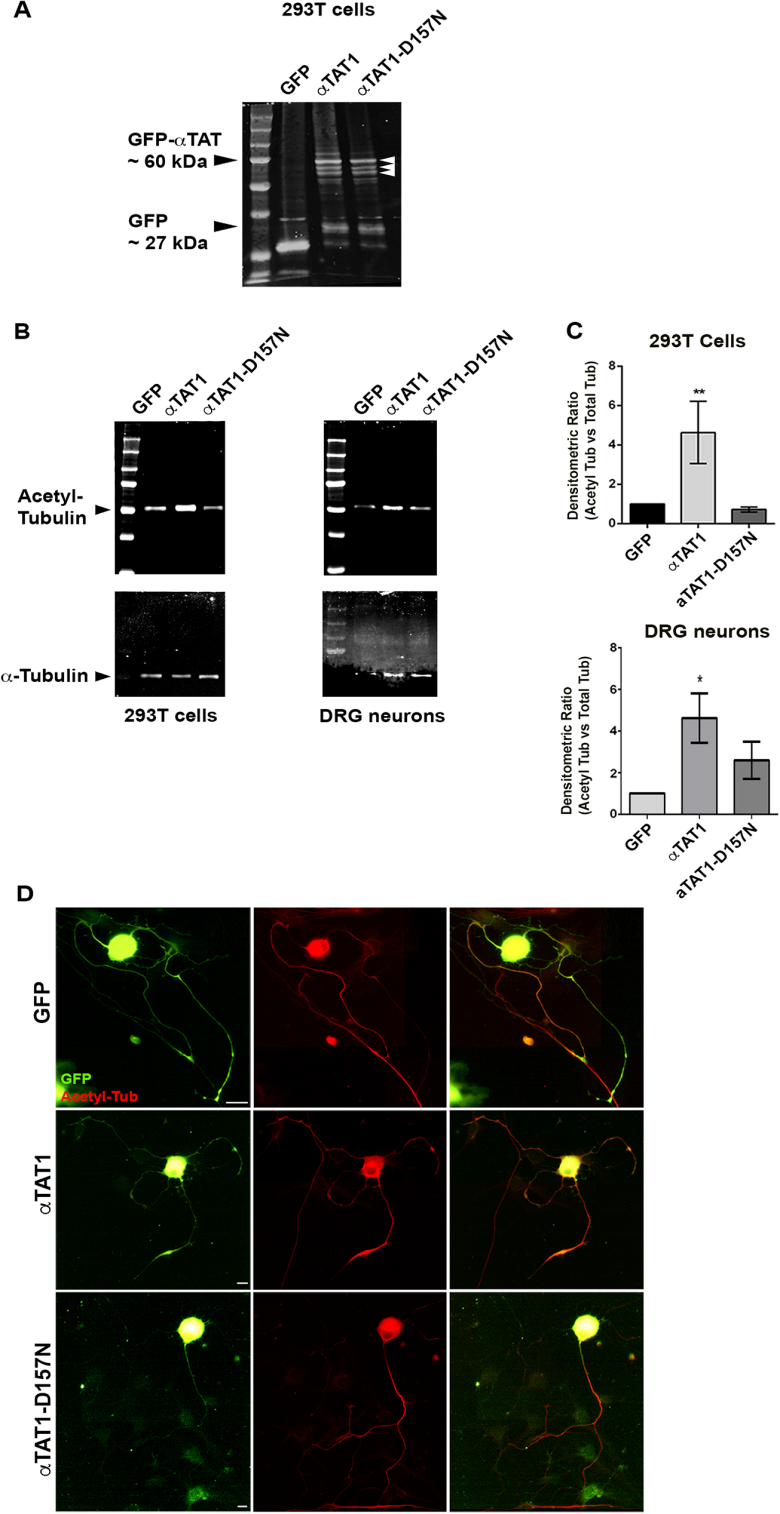
αTAT1 overexpression increases alpha-tubulin acetylation but αTAT1-D157N does not significantly increase alpha-tubulin acetylation in 293T cells and DRG neurons. **A**: 293T cells were infected with lentiviral constructs encoding GFP, αTAT1 and αTAT1-D157N, all of which are tagged with GFP. Cells were collected and run on Western Blots before being probed with anti-GFP antibody. The αTAT1 constructs migrated at around 60 kDa (arrowheads). **B**: 293T cells and DRG neurons infected with GFP, αTAT1 and αTAT1-D157N were run on Western Blots and probed with 6-11B-1 antibody for acetylated alpha-tubulin and anti-alpha-tubulin antibodies. **C**: The densitometric ratio of acetylated alpha-tubulin compared to alpha-tubulin was measured for GFP, αTAT1 and αTAT1-D157N for each blot. In 293T cells, the densitometric ratios of αTAT1 and αTAT1-D157N were 4.63 ± 1.58 and 0.72 ± 0.12 respectively relative to GFP. The ratios were normalized to GFP, serving as the control. In cultured DRG neurons, the densitometric ratios of αTAT1 and αTAT1-D157N were 4.62 ± 1.20 and 2.60 ± 0.89 respectively relative to GFP. (** denotes p<0.01, * denotes p<0.05; n = 4; bars show SEM). **D**: DRG neurons were infected with GFP, αTAT1 or αTAT1-D157N lentivirus and grown for two days before being replated and grown for another day. Neurons were fixed and labeled with GFP and 6-11B-1 antibodies, (scale bar, 10 μm).

To confirm the expression and function of lentiviruses, GFP, αTAT1 or αTAT1-D157N was infected into 293T cells. All expressed proteins had a GFP tag. Cells were left to grow for 48 hrs until a maximum level of GFP fluorescence could be seen inside the cells, before lysis and collection. The cell lysates were loaded, run on gels for Western Blotting and probed with anti-GFP antibody. The results show strong expression of GFP, marked by a 27 kDa band. αTAT1 and αTAT1-D157N are represented by bands that migrate at just over 60 kDa (Fig 1A). This is in accordance with the literature, which indicates that without a tag, αTAT1 migrates at around 30 kDa and 37 kDa [21, 24]. A variety of other bands were detected on the gel, possibly due to non-specific antibody labeling, or proteolysis during sample preparation.

Studies have also shown that αTAT1 acetylates microtubules in DRG neurons, CHO cells and NIH3T3 cells [20]. We set out to confirm that in adult DRG neurons, αTAT1 acetylates alpha-tubulin after overexpressing the construct using lentivirus. Dissociated DRG neurons were cultured and infected with GFP, αTAT1 or αTAT1-D157N lentivirus. Following infection, neurons were grown for a further three days to allow maximum gene expression, as visualized by GFP fluorescence. Neurons were lysed and collected for Western blotting before being probed for acetylated alpha-tubulin and alpha-tubulin. To look at the effects of these genes on acetylation in non-neuronal cells 293T cells were also infected with the same lentiviruses, allowed to grow for 48 hrs to reach maximum gene expression and collected for Western blotting. 293T cells were also probed for acetylated alpha-tubulin and alpha-tubulin. In both 293T cells and in adult DRG cultured neurons, infection with αTAT1 resulted in an increased ratio of acetylated alpha-tubulin (Fig 1B). In 293T cells, the densitometric ratio of acetylated alpha-tubulin in αTAT1-infected cells was significantly increased relative to that of cells expressing GFP (Fig 1C). There was no significant increase in acetylated alpha-tubulin as a result of αTAT1-D157N expression in 293T cells. In adult DRG neurons, the densitometric ratio of cultures infected with αTAT1 was also significantly increased relative to that of cell expressing GFP (Fig 1C). There was a smaller increase in acetylated alpha-tubulin as a result of αTAT1-D157N expression in DRG neurons but this was not statistically significant. The fact that only around 30% of all cultured neurons were infected and that cell lysates contained mixtures of neurons and non-neuronal cells suggests that the data from Western Blotting was not sufficient to enable a clear interpretation of the effects of the constructs on axonal acetylation. To gain a better picture, we proceeded to examine individual neurons in culture that were infected.

To observe expression of αTAT1 and αTAT1-D157N, dissociated DRG neurons were infected with lentiviruses. Neurons were infected with lentivirus, replated and allowed to grow for a further two days before being fixed and labeled with GFP and 6-11B-1 antibodies. Similar to GFP expression, αTAT1 was distributed in the cell body, along the entire axon and at the growth cones. Expression of αTAT1 was sometimes punctate and was concentrated in the cell body and proximal part of the axon (Fig 1D). Neurons infected with αTAT1-D157N showed a similar pattern, with higher levels in the proximal part of the axon (Fig 1D). To observe the expression of HDAC5, DRG neurons were infected with HDAC5-Nuc and HDAC5-Cyt constructs. HDAC5-Cyt has serine-to-aspartate substitutions at residues 259 and 498 and has been reported to be exported out of the cell body into the axon after an injury response while HDAC5-Nuc has serine-to aspartate substitutions at residues 259, 280, and 498 and has been reported to stay inside the cell body [16]. Neurons were fixed after growth for two days before being labeled with GFP and βετα-III-tubulin antibody. Our studies confirmed previous findings that HDAC5-Cyt is predominantly localized to the axonal cytoplasm while HDAC5-Nuc is predominantly expressed in the nucleus in culture (Panel A in S1 Fig). Comparison of axon outgrowth for neurons expressing HDAC5-Cyt with HDAC5-Nuc showed no significant differences compared to neurons expressing GFP, either in longest axon lengths or total axon lengths (Panel B in S1 Fig). There were also no differences in axon lengths between neurons expressing HDAC5-Cyt and HDAC5-Nuc. It was decided that HDAC5-Cyt would be used for all subsequent experiments since this construct could be expressed in the axonal cytoplasm where it may have an effect on axonal microtubules.

### αTAT1 increases acetylated alpha-tubulin while HDAC5 decreases acetylated alpha-tubulin at the tips of DRG axons

**Fig 2:**
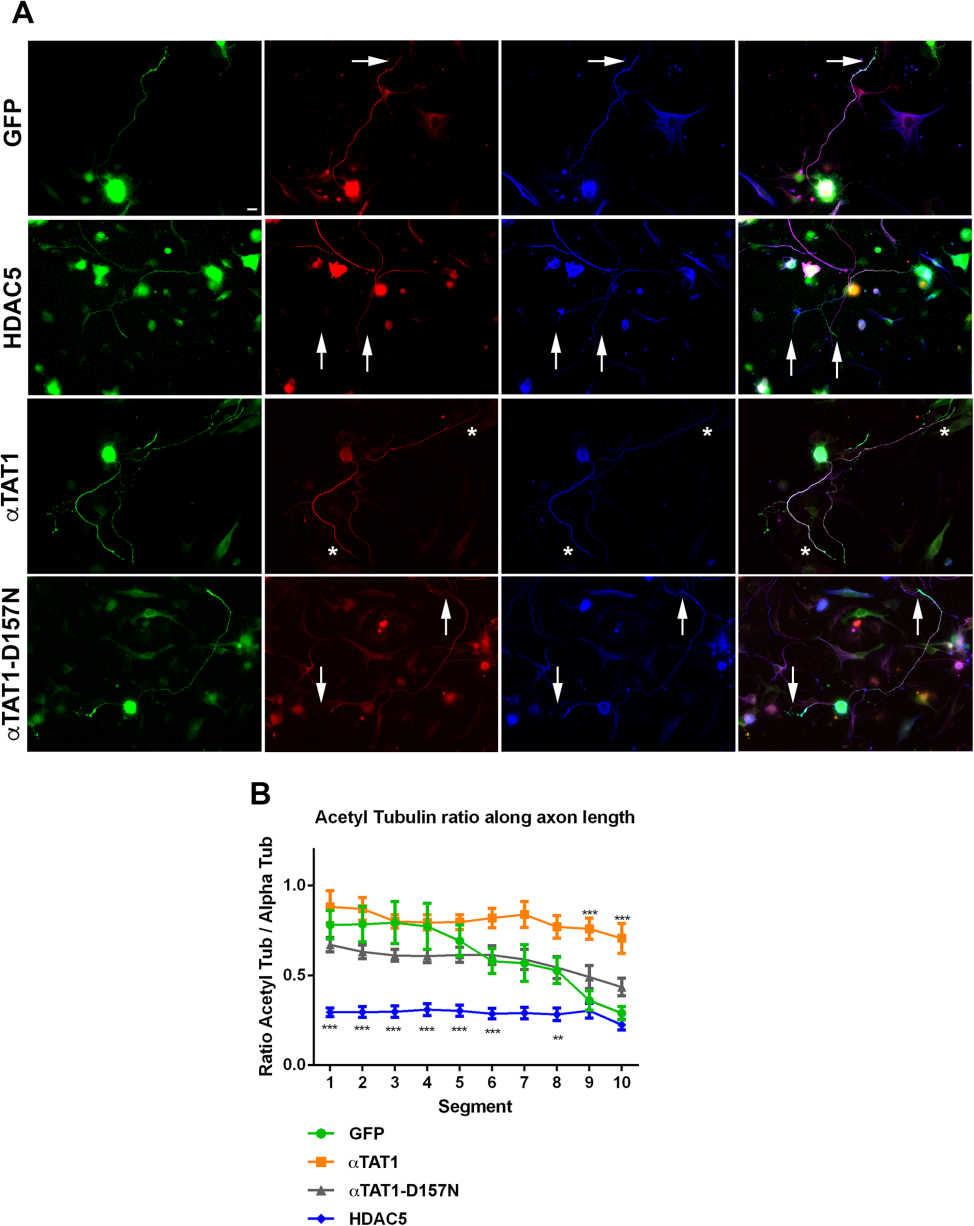
αTAT1 increases acetylated alpha-tubulin in distal axons and HDAC5 decreases acetylated alpha-tubulin along the length of the axon. DRG neurons were infected with GFP, HDAC5, αTAT1 or αTAT1-D157N. Neurons were allowed to express the genes for two days before replating and fixation. Neurons were labeled with anti-GFP, 6-11B-1 for acetylated alpha-tubulin and alpha-tubulin antibodies. **A**: Representative images of neurons infected with each construct and labeled with anti-GFP antibody (green), acetylated alpha-tubulin (red) and alpha-tubulin antibody (blue). Panels on the far right column show merged images. Neurons expressing GFP, αTAT1-D157N, or HDAC5 show diminished labeling of acetylated alpha-tubulin at the axonal tips (arrows). However, neurons treated with αTAT1 show acetylated alpha-tubulin extending into the axon tips (asterix), (scale bar, 10 μm) **B**: The ratio of acetylated alpha-tubulin against the total alpha-tubulin (red / blue pixel intensity) was measured along 10 equally spaced segments spanning the length of the axons, with 1 being closest to the cell body and 10 representative of the axon before the growth cone. In neurons infected with αTAT1, the acetylated alpha-tubulin ratio values at Segments 9 and 10 (0.75 ± 0.07 and 0.75 ± 0.08 respectively) were significantly higher than in neurons infected with GFP (0.39 ± 0.05 and 0.31 ± 0.03 respectively) at the same segments (p<0.001***). In neurons infected with HDAC5 the acetylated alpha-tubulin ratio values were significantly lower at many segments along the axon (Segment 1: 0.29 ± 0.02, Segment 2: 0.29 ± 0.03, Segment 3: 0.30 ± 0.03, Segment 4: 0.31 ± 0.03, Segment 5: 0.30 ± 0.03, Segment 6: 0.28 ± 0.03, Segment 7: 0.29 ± 0.03, Segment 8: 0.28 ± 0.03) relative to the same segments in neurons infected with GFP (Segment 1: 0.78 ± 0.08, Segment 2: 0.78 ± 0.10, Segment 3: 0.79 ± 0.12, Segment 4: 0.77 ± 0.13, Segment 5: 0.69 ± 0.09, Segment 6: 0.58 ± 0.07, Segment 7: 0.57 ± 0.10, Segment 8: 0.52 ± 0.07), (p<0.001***, segments 1–3, 5, 8 and p<0.01**, segments 4, 6, 10), (GFP: n = 13, αTAT1: n = 16, αTAT1-D157N: n = 17, HDAC5: n = 14; error bars represent SEM).

In axons acetylated alpha-tubulin exhibits a proximal (high) to distal (low) gradient. The levels of alpha-tubulin acetylation are lowest at the tips of axons where microtubules are most dynamic [12, 13, 29]. To assess the extent of alpha-tubulin acetylation across the axon, dissociated DRG neurons, infected with GFP, HDAC5, αTAT1 or αTAT1-D157N, replated, fixed and labeled for acetylated alpha-tubulin and for total alpha-tubulin. Only GFP positive neurons were examined. Neurons expressing GFP showed high levels of acetylated alpha-tubulin in the proximal segments and a progressive decrease in acetylated alpha-tubulin toward the distal segments, as previously described [12, 13, 29], (Fig 2A and 2B). Neurons infected with αTAT1 showed relatively high levels of acetylated alpha-tubulin ratios along all segments of the axon. In neurons infected with αTAT1, the acetylated alpha-tubulin ratio values at Segments 9 and 10 were significantly higher than in neurons infected with GFP at the same segments (Fig 2B). Interestingly, in neurons infected with αTAT1-D157N, axons also displayed a progressive decrease in acetylated alpha-tubulin towards the distal end of axons, similar to axons infected with GFP. This suggests that αTAT1-D157N does not play as strong a role in microtubule acetylation as wild-type αTAT1 in axons. In neurons infected with HDAC5, where HDAC5 was expressed in the cytoplasm, the acetylated alpha-tubulin ratio values were significantly lowered in most of the segments along the axon (segments 1 to 8) relative to neurons infected with GFP. In these neurons the values for acetylated alpha-tubulin were less than half that of neurons infected with GFP.

### Overexpression of αTAT1, but not HDAC5, increases axonal regeneration in neurons after replating

**Fig 3:**
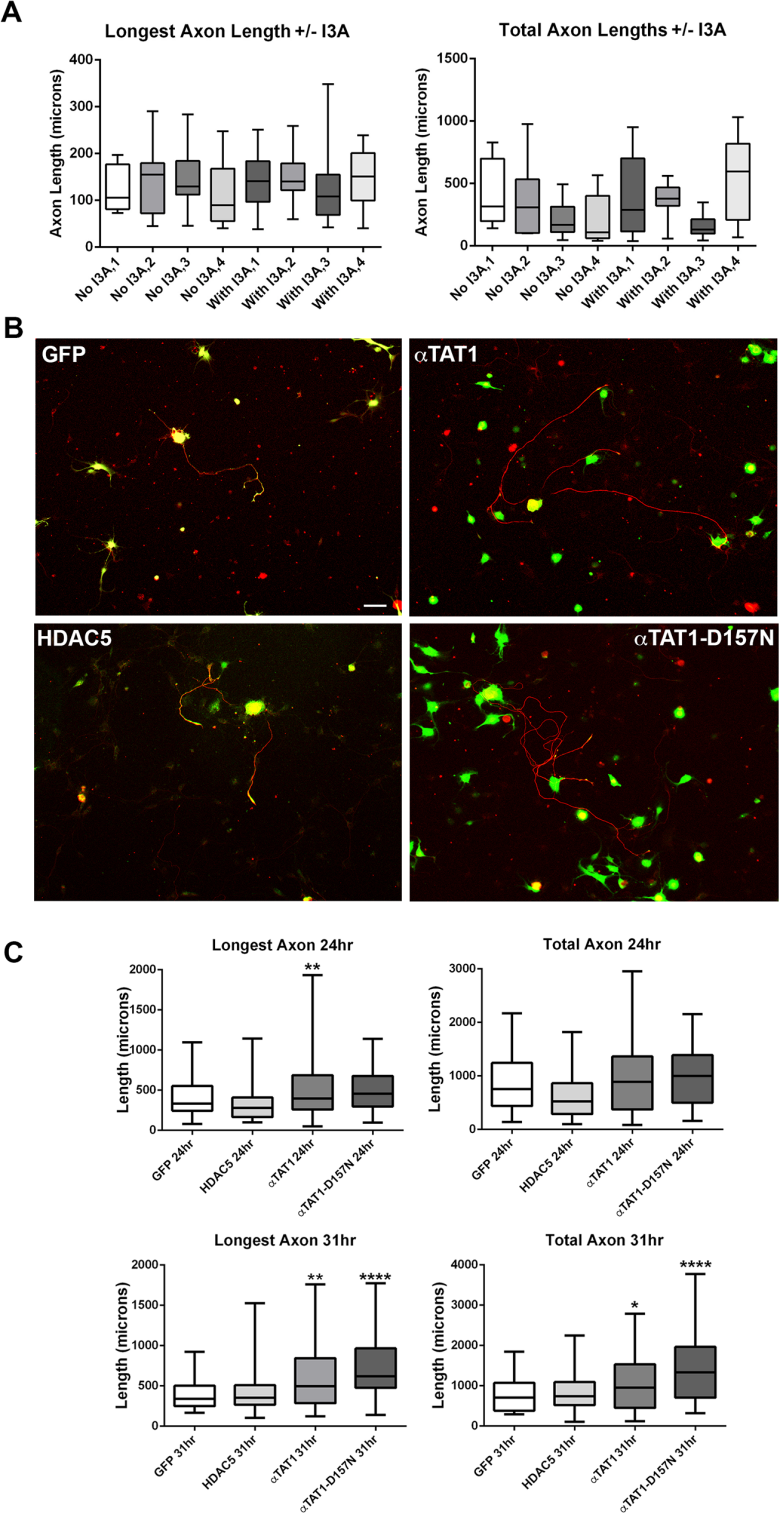
αTAT1 and αTAT1-D157N infection of DRG neurons increases axon lengths while HDAC5 does not significantly affect axon lengths. Adult DRG neurons were infected with GFP, HDAC5, αTAT1 or αTAT1-D157N and grown for two days until genes were expressed. Neurons were replated and left to grow for another 24 hrs or 31 hrs before being fixed and labeled with anti-GFP antibody (green) and βετα-III-tubulin (red). **A**: Box graphs showing quantification of the mean axon lengths for four separate neuronal cultures infected with HDAC5 and treated with or without I3A before fixation. There were no significant differences in longest axon lengths relative to neurons without I3A (mean axon lengths of neurons with no I3A were: 121.8 ± 48.76 μm; 140.1 ± 76.15 μm; 143.7 ± 64.03 μm; 111.1 ± 69.78 μm and with I3A were: 139.4 ± 14.94 μm; 144.5 ± 15.77 μm; 127.4 ± 28.36 μm; 148.9 ± 16.3 μm). There were also no significant differences in total axon lengths in neurons treated with or without I3A (mean total axon lengths of neurons with no I3A were: 403.1 ± 95.72 μm; 345.8 ± 102.3 μm; 203.8 ± 42.64 μm; 11.7 ± 67.39 μm and with I3A were: 363.8 ± 77.55 μm; 358.7 ± 42.29 μm; 160.3 ± 32.15 μm; 527.2 ± 88.62 μm). **B**: Representative images of neurons infected with various constructs (scale bar, 20 μm). **C**: Box graphs showing quantification of mean axon lengths for neurons expressing each gene, categorized into “Longest Axon” or “Total Axon”. At 24 hrs after replating, neurons infected with GFP, HDAC5, αTAT1 and αTAT1-D157N grew their longest axons to 397.8 ± 27.45 μm, 392.4 ± 34.72 μm, 510.7 ± 59.94 μm and 499.1 ± 30.61 μm respectively. Neurons infected with GFP, HDAC5, αTAT1 and αTAT1-D157N grew to a total length of 865 ± 70.25 μm, 639.8 ± 72.05 μm, 999.9 ± 116.8 μm and 993.8 ± 64.48 μm respectively. At 31 hrs after replating, Neurons transduced with GFP, HDAC5, αTAT1 and αTAT1-D157N grew their longest axons to 387.4 ± 28.67 μm, 410.2 ± 29.99 μm, 592.4 ± 48.41 μm and 687.9 ± 51.76 μm, respectively. Neurons infected with GFP, HDAC5, αTAT1 and αTAT1-D157N grew total axon lengths to 746.9 ± 59.24 μm, 845.2 ± 60.96 μm, 1069 ± 99.96 μm and 1455 ± 135.4 μm respectively. Boxes show the maximum, mean and minimum measurements in each group (24 hrs—GFP: n = 57, HDAC5: n = 37 αTAT1: n = 37, αTAT1-D157N: n = 61, Longest Axon Lengths: p<0.01** for αTAT1), (31 hrs–GFP: n = 41, HDAC5: n = 58, αTAT1, n = 51, αTAT1-D157N: n = 42, Longest Axon Lengths: p<0.01** for αTAT1 and p<0.001*** for αTAT1-D157N, Total Axon Lengths: p<0.05* for αTAT1 and p<0.001*** for αTAT1-D157N). Bars show SEM.

Adult DRG neurons were infected with GFP, HDAC5, αTAT1 or αTAT1-D157N and left to grow for two days before being replated on coverslips. Cultures infected with HDAC5 were additionally treated with I3A for 2 hrs after replating before media was washed off and replenished. I3A is a PKCμ activator which phosphorylates HDAC5 and exports it from the nucleus into the axonal cytoplasm, where it can deacetylated microtubules in the axon [15, 18, 28]. Addition of I3A to these neurons showed no significant effects on axon lengths compared to neurons with no treatment of I3A (Fig 3A). Subsequently, all cultures infected with HDAC5 and GFP virus were treated with I3A before fixation and axon quantification.

Neurons were fixed after 24 hrs and 31 hrs following replating and stained with β-III-tubulin. The lengths of all axons expressing GFP in each group was measured (Fig 3B). At 24 hrs after replating, there were no significant differences between any of the groups apart from cultures treated with αTAT1. Axon lengths were categorized into two groups: the longest axon of the neuron (longest axon length) or the total lengths of all processes of the neuron (total axon lengths). Neurons infected with GFP and αTAT1 grew significantly longer axons compared to those expressing GFP (Fig 3B). Axons from αTAT1-D157N treated neurons were longer, but were not statistically significantly. Measurements of the total axon lengths from all groups revealed no statistically significant differences. Axons of neurons expressing HDAC5 showed a decrease in mean axon lengths compared to axons expressing GFP but it was not statistically significant (Fig 3C).

Thirty-one hrs after replating, αTAT1 and αTAT1-D157N-infected neurons grew axons that were significantly longer than neurons transduced with GFP. The longest axon lengths expressing αTAT1 and αTAT1-D157N were significantly longer than those expressing GFP or HDAC5. The total lengths of axons expressing αTAT1 and αTAT1-D157N were significantly longer than those expressing GFP or HDAC5, (Fig 3C).

### Overexpression of HDAC5 and αTAT1 by sciatic nerve axons failed to improve axonal regeneration

**Fig 4:**
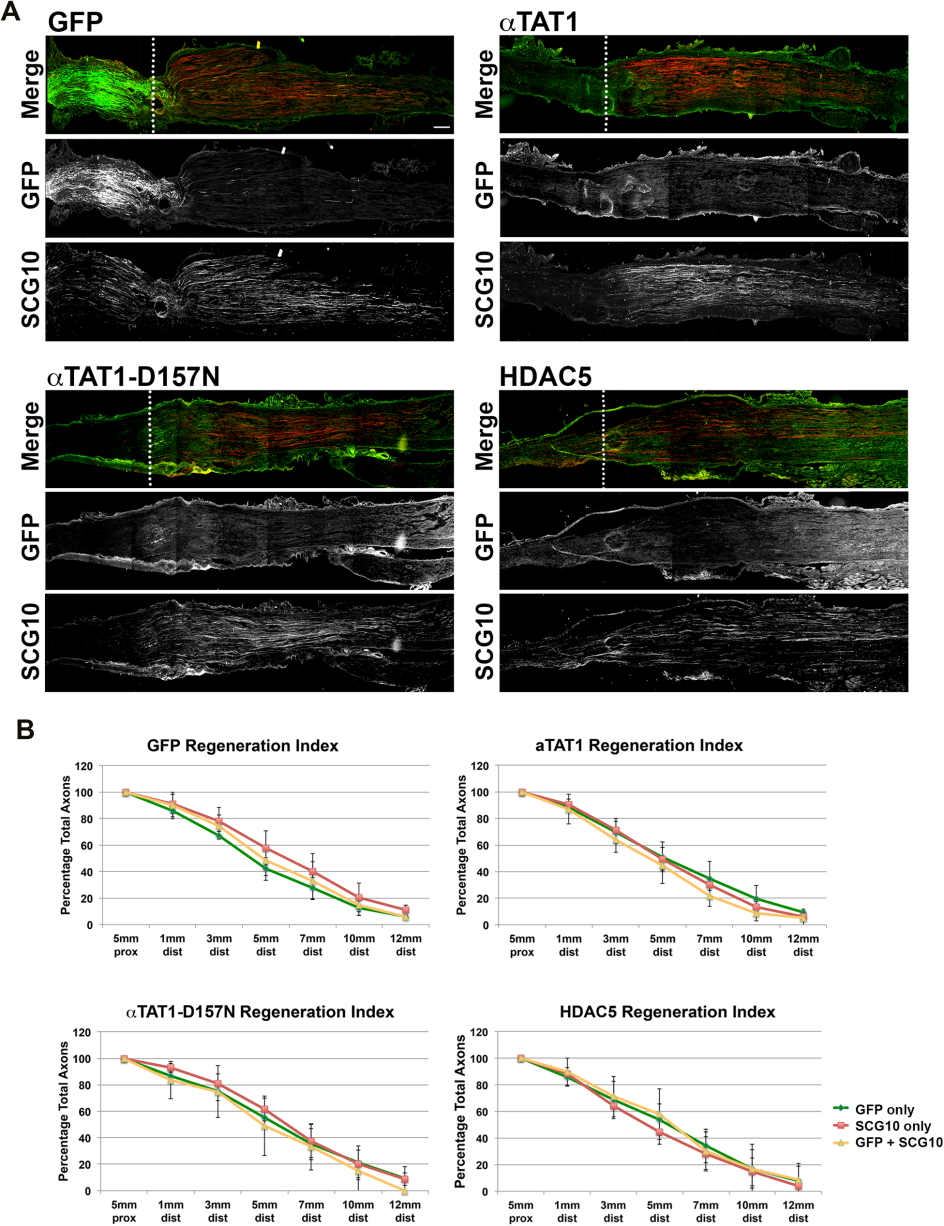
Axonal regeneration after Sciatic nerve crush is not augmented by αTAT1, αTAT1-D157N or HDAC5. Adult rat sciatic nerves were injected with lentiviruses to express GFP, αTAT1, αTAT1-D157N or HDAC5. After gene expression, sciatic nerves were crushed and axons were left to recover for 5 days before animals were sacrificed. Sciatic nerves were sectioned longitudinally and stained with anti-GFP antibody (green) and with SCG10 antibody (red). **A**: Representative images of sciatic nerves showing crush site (dashed line) in the top panel for a merged image, infected axons marked by GFP expression (GFP, middle panel) and regenerating axons in the distal segment (SCG10, bottom panel). Separate GFP and SCG10 channels are displayed for each merged image (scale bar, 500 μm). **B**: Regeneration index of axons at various distances before and after the lesion site comparing infected axons (GFP only), regenerating axons (SCG10 only), and axons infected and regenerating (GFP + SCG10). No significant differences in axonal regeneration index between any of the groups observed between 5 mm proximal, 1 mm distal, 3 mm distal, 5 mm distal, 7 mm distal or 12 mm distal (bars show SEM values).

To test the effect of HDAC5 and αTAT1 on axonal regeneration, we injected adult rat sciatic nerves with lentivirus-driven GFP, HDAC5, αTAT1 or αTAT1-D157N. Sciatic nerves were crushed and left to recover for five days before animals were perfused. Sciatic nerves were collected and sectioned longitudinally before being labeled with GFP and SCG10 antibodies. SCG10 recognizes Stathmin, a microtubule associated protein that is expressed abundantly in regenerating axons of the sciatic nerves [30]. For each sciatic nerve, the total numbers of transduced axons (GFP-positive) and the total numbers of regenerating axons (SCG10 positive axons) at various distanced from the lesion site were counted separately. To determine the number of transduced axons actively undergoing regeneration we counted the number of axons labeled by both GFP and SCG10. Samples were taken at various distances proximal and distal to the lesion site and a regeneration index was calculated (see Materials and Methods). No observable differences could be seen for regenerating axons infected with any of the constructs relative to GFP controls (Fig 4A). No significant differences were observable between the number of transduced axons (GFP only), or the total number of axons regenerating (SCG10 only) at each distance for any of the rats. Similarly, no significant differences were observed between any of the groups for axons that were both transduced and regenerating (GFP + SCG10; Fig 4B).

## Discussion

This study investigated the effects of αTAT1 and HDAC5 on axonal growth and regeneration in adult DRG neurons *in vitro* and *in vivo*. Overexpression of αTAT1 increased acetylated alpha-tubulin along the length of axons in adult DRG neurons. Expression of αTAT1 and the catalytically inactive mutant αTAT1-D157N increased axon lengths of DRG neurons *in vitro*. Expression of HDAC5, known to deacetylate alpha-tubulin when transported into the axon, had relatively little effect on axon lengths in our experiments. When these constructs were overexpressed in the rat sciatic nerve *in vivo*, no improvement in axonal regeneration was observed relative to sciatic nerves that expressed the GFP control. Our results are the first to show that overexpression of αTAT1 can increase the axonal length of DRG neurons in the culture dish. A recent study showed that αTAT1 does not play a role in axonal growth of NGF-responsive DRG neurons in mice [25]. However, our results differ since the neuronal cultures were grown with no neurotrophins in the media. Our observations may be the result of non-NGF responsive neurons.

αTAT1 has been shown to destabilize microtubules independently of its acetylation ability by directly binding to microtubules in NIH3T3 cells [20]. To test whether αTAT1 could promote axonal regeneration independently of its catalytic activity, we overexpressed the αTAT1-D157N mutant in DRG neurons. Our Western blot data showed that this mutant construct could not cause a significant increase in alpha-tubulin acetylation and our immunolabeling data showed that it did not have a significant effect on increasing microtubule acetylation in the distal tips of axons. This finding confirms previous data in PtK2 cells [24], mouse embryonic fibroblast cells [21] and mechanosensitive neurons in mice [25]. However, like the αTAT1 wild type construct, αTAT1-D157N did promote DRG axonal growth *in vitro*. Part of the explanation could be that mutating the D157 residue does not fully disrupt catalytic activity. Since αTAT1 requires highly conserved residues, D157, Q58 and C120 for its catalytic activity, to accept Acetyl CoA as a donor for the acetyl group [31], the D157N mutation alone may not be sufficient to disrupt the αTAT1 catalytic activity completely in all neurons. It is unknown whether the other two residues are essential for neuronal microtubule acetylation. Thus, residual αTAT1 catalytic activity may still occur in neuronal cultures, resulting in increased axonal growth. On the other hand, since the Western blots showed αTAT1-D157N infection did not increase overall acetylated tubulin, this suggests that αTAT1 may induce increased axonal growth independently of catalytic activity, *in vitro*. How this regulation happens would require further investigation.

Our sciatic nerve crush experiments showed that overexpressing αTAT1 failed to promote axonal regeneration. Out of the proportion of axons that were infected and were GFP positive, the number of regenerating axons, labeled with SCG10, did not significantly increase distal to the lesion site in animals infected with αTAT1 or HDAC5 compared to those infected with GFP alone. The same was true for axons labeled with both GFP and SCG10. This suggests that αTAT1 does not play a role in axonal regeneration, at least in sciatic nerves. Our investigation also showed that HDAC5 overexpression does not improve axonal regeneration in cultured DRG neurons or in sciatic nerves after injury. Previous studies have shown that HDAC5 plays an important role in axonal regeneration, following an injury-induced calcium response that shuttles it from the nucleus to the cytoplasm [15, 16, 18]. It was reported that HDAC5, when expressed only in the cytoplasm and not the nucleus could facilitate regeneration but HDAC5 expressed in the nucleus alone could not play a role. While we confirmed that HDAC5 can localize separately in the nucleus and in the cytoplasm in neurons (S1 Fig), we did not find that expression of HDAC5 solely in the cytoplasm had an effect on improving axonal regeneration. Furthermore, contrary to previous literature, application of I3A, a PKC phosphorylation activator known to export HDAC5 from the nucleus to the cytoplasm, did not affect axon lengths of neurons infected with HDAC5. Unlike previous experiments that used DRG neurons from mice with a prior sciatic nerve injury, our experiments used adult rats that did not receive a prior injury. Our experiments did not use an axotomy model to lesion the axons *in vitro* and used a replating protocol instead. Such differences in the protocol may have altered the impact of HDAC5 on regeneration. Since previous studies have shown that knockdown of HDAC5 is detrimental to sciatic nerve regeneration and to growth cone dynamics [15, 16], it remains to be seen how HDAC5 could affect axonal regeneration. Axons in the sciatic nerve could respond differently to injury compared to axons in the central nervous system. Previous studies have shown that the optic nerve undergoes a different pattern of regeneration associated gene expression [18] and that overexpression of histone acetylation proteins, such as p300, can promote optic nerve regeneration [7]. Future investigations could be geared towards investigating whether overexpression of αTAT1 or HDAC5 improve axonal regeneration after lesions in the central nervous system as opposed to the peripheral nervous system.

Recently it was shown that αTAT1 acetylates Lysine 40 on alpha-tubulin by end-entry of the microtubule lumen and through nicks and openings in the microtubule lattice [32]. Following axotomy in neurons, a more disturbed microtubule lattice could provide a platform for more αTAT1 to enter the lumen and increase acetylated patches of microtubules in the region of axonal damage. It is possible that αTAT1 could play a destabilizing role for microtubules by its microtubule binding activity [20]. αTAT1 also shares a structural homology with another acetyltransferase, Gcn5 related N-acetyltransferase and belongs in the HAT family which includes ELP3, GNC5 and an N-α-acetyltransferase [33, 34]. These HATs may cause minor levels of alpha-tubulin acetylation that could affect axonal regeneration.

While our data regarding the distribution of microtubule acetylation agrees with previous findings, that acetylated alpha-tubulin along the length of the axon decreases towards the distal end of growing DRG neurons [10], we did not observed evidence to suggest that alpha-tubulin acetylation is directly involved with axonal regeneration in adult neurons. The possibility exists that the graded decrease of alpha-tubulin acetylation along the length of the microtubule belongs to part of a greater posttranslational modification “code” which would enable microtubules to become more labile during growth [11, 12, 35]. Other microtubule associated proteins, such as severing proteins katanin and spastin could also be at play to regulate cycles of microtubule polymerization to influence axonal growth [36]. Future studies will be required to determine whether other forms of microtubule posttranslational modification or microtubule associated proteins could play a role in axonal regeneration.

## Supporting information

S1 Data (Supporting information data for Fig 1): Densitometric ratio for Western blots. Densitometric ratio of Western blots comparing acetylated tubulin against total tubulin in cells infected with GFP, αTAT1 or αTAT1-D157N.
(XLSX)

S2 Data (Supporting information data for Fig 2): Densitometric ratio of axonal tubulin fluorescence. Densitometric ratio of fluorescence intensity along the axon length comparing acetylated tubulin against total tubulin in neurons infected with GFP, αTAT1, αTAT1-D157N or HDAC5.
(XLSX)

S3 Data (Supporting information data for Fig 3): Axon lengths. Axon lengths of neurons infected with GFP, αTAT1, αTAT1-D157N or HDAC5. Also comparison of axon lengths from neurons treated with I3A or without I3A.
(XLSX)

S4 Data (Supporting information data for Fig 4): Axon regeneration index. Regeneration index of sciatic nerves after crush, infected with GFP, αTAT1, αTAT1-D157N or HDAC5.
(XLSX)

S1 Fig HDAC5 constructs can be localized to different compartments in the nucleus and cytoplasm but overexpression does not affect axon length. Adult rat DRG neurons were infected with GFP, HDAC5-Nuc or HDAC5-Cyt and allowed to express the genes before being replated and fixed. Neurons were labeled with anti-GFP antibody (green) and βετα-III-tubulin (red). **A**: Representative images show expression patterns of GFP, HDAC5-Nuc and HDAC5-Cyt before addition of any drug (scale bar, 10 μm). **B**: Box graphs showing quantification of mean axon lengths for GFP, HDAC5-Cyt and HDAC5-Nuc, categorized into “Longest Axon” or “Total Axon”. Boxes show the maximum, mean and minimum measurements in each group (n = 12, Bars show SEM). No significant differences observed between any of the groups.
(TIF)

S1 File (Supporting information data for S1 Fig): Axon lengths. Axon lengths of neurons infected with GFP, HDAC5-Cyt or HDAC5-Nuc.
(XLSX)
