# Supplementary figures and images for: Effects of αTAT1 and HDAC5 on axonal regeneration in adult neurons
Source: PLoS One. 2017 May 15;12(5):e0177496. doi: 10.1371/journal.pone.0177496 (PMC5432171; doi:10.1371/journal.pone.0177496)

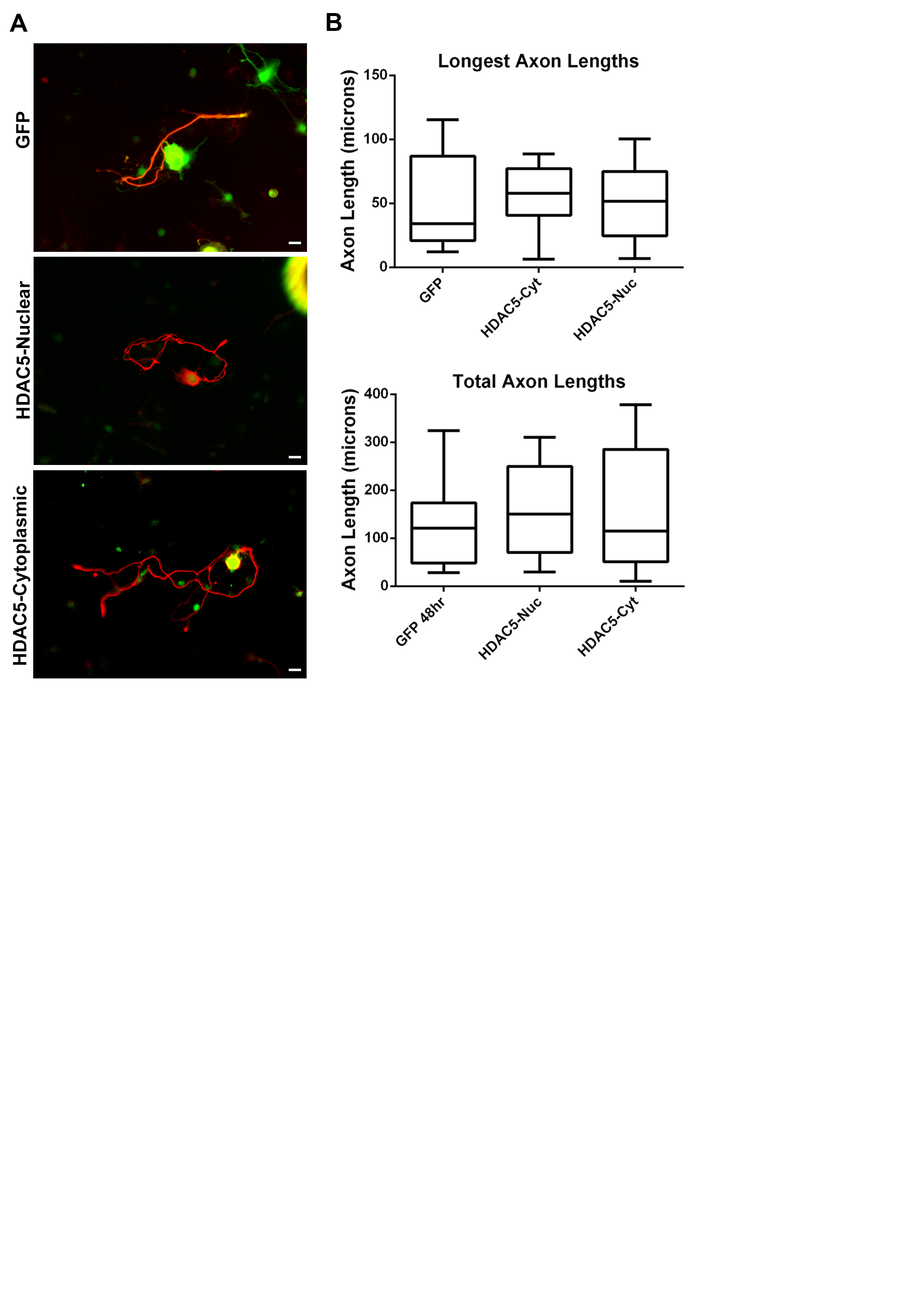

Supplement: S1 Fig — Adult rat DRG neurons were infected with GFP, HDAC5-Nuc or HDAC5-Cyt and allowed to express the genes before being replated and fixed. Neurons were labeled with anti-GFP antibody (green) and βετα-III-tubulin (red). A: Representative images show expression patterns of GFP, HDAC5-Nuc and HDAC5-Cyt before addition of any drug (scale bar, 10 μm). B: Box graphs showing quantification of mean axon lengths for GFP, HDAC5-Cyt and HDAC5-Nuc, categorized into “Longest Axon” or “Total Axon”. Boxes show the maximum, mean and minimum measurements in each group (n = 12, Bars show SEM). No significant differences observed between any of the groups. (TIF) [file pone.0177496.s005.tif]
